# Dark Rearing Does Not Alter Developmental Retinoschisis Cavity Formation in Rs1 Gene Knockout Rat Model of X-Linked Retinoschisis

**DOI:** 10.3390/genes16070815

**Published:** 2025-07-11

**Authors:** Zeljka Smit-McBride, In Hwan Cho, Ning Sun, Serafina Thomas, Paul A. Sieving

**Affiliations:** 1 Department of Ophthalmology, Eye Center, UC Davis School of Medicine, Sacramento, CA 95817, USA; zsmcbride@ucdavis.edu (Z.S.-M.); ophcho@ucdavis.edu (I.H.C.); 2 Vitreoretinal Research Lab, UC Davis School of Medicine, Davis, CA 95616, USA; 3 Department of Ophthalmology, College of Medicine, Soonchunhyang University, Cheonan 31151, Republic of Korea; 4 Department of Cell Biology & Human Anatomy, UC Davis, Davis, CA 95616, USA

**Keywords:** retinoschisin, RS1, dark rearing, X-linked retinoschisis, XLRS, rat retina disease model, retinoschisis, retinoschisin, glia cells, MGC, retina development

## Abstract

Background/Objective: The *Rs1* exon-1-del rat (Rs1KO) XLRS model shows normal retinal development until postnatal day 12 (P12) when small cystic spaces start to form in the inner nuclear layer. These enlarge rapidly, peak at P15, and then collapse by P19. These events overlap with eye opening at P12–P15. We investigated whether new light-driven retinal activity could contribute to the appearance and progression of schisis cavities in this rat model of XLRS disease. Methods: For dark rearing (D/D), mating pairs of Rs1KO strain were raised in total darkness in a special vivarium at UC Davis. When pups were born, they were maintained in total darkness, and eyes were collected at P12, P15, and P30 (n = 3/group) for each of the D/D and cyclic light-reared 12 h light–12 h dark (L/D) Rs1KO and wild-type (WT) littermates. Eyes were fixed, paraffin-embedded, and sectioned. Tissue morphology was examined by H&E and marker expression of retinoschisin1 (Rs1), rhodopsin (Rho), and postsynaptic protein 95 (Psd95) by fluorescent immunohistochemistry. H&E-stained images were analyzed with ImageJ version 1.54h to quantify cavity size using the “Analyze Particles” function. Results: Small intra-retinal schisis cavities begin to form by P12 in the inner retina of both D/D and L/D animals. Cavity formation was equivalent or more pronounced in D/D animals than in L/D animals. We compared Iba1 (activation marker of immune cells) distribution and found that by P12, when schisis appeared, Iba1^+^ cells had accumulated in regions of schisis. Iba1^+^ cells were more abundant in Rs1KO animals than WT animals and appeared slightly more prevalent in D/D- than L/D-reared Rs1KO animals. We compared photoreceptor development using Rho, Rs1, and Psd95 expression, and these were similar; however, the outer segments (OSs) of D/D animals with Rho labeling at P12 were longer than L/D animals. Conclusions: The results showed that cavities formed at the same time in D/D and L/D XLRS rat pups, indicating that the timing of schisis formation is not light stimulus-driven but rather appears to be a result of developmental events. Cavity size tended to be larger under dark-rearing conditions in D/D animals, which could be due to the decreased rate of phagocytosis by the RPE in the dark, allowing for continued growth of the OSs without the usual shedding of the distal tip, a key mechanism behind dark adaptation in the retina. These results highlight the complexity of XLRS pathology; however, we found no evidence that light-driven metabolic activity accounted for schisis cavity formation.

## 1. Introduction

Environmental light exposure is important for the normal maturation of the retina of mice and rats, and it is known that conditions of light exposure can modify the natural history of inherited retinal degeneration in animal models [1,2,3,4,5]. It is also reported that light deprivation, by dark rearing or by suturing the eyelids shut, retards light-dependent maturation of visual pathway activity in the proximal retina involving retinal ganglion cells [6,7] and visual parts of the brain [3].

In working with *Rs1* exon-1 deletion knockout mice [2], a model for X-linked retinoschisis, we previously demonstrated that the amount of daily cyclic light exposure altered the degree of XLRS structural and functional pathology at a young age [1]. Rs1KO mice housed in low, 20-lux lighting had fewer retinal cavities, and the inner retinal structure was more organized than that of Rs1KO mice reared at 300 lux. This was surprising, as neither 20 nor 300 lux is at photochemical retinal-damaging light levels [8,9]. This indicated an unusual interaction of low-level light exposure with the mature Rs1KO retina.

We subsequently generated a rat *Rs1* exon-1-del Rs1KO model of XLRS pathology [10], homologous to the *Rs1* exon-1-del Rs1KO mouse model, and have been studying the developmental course of schisis cystic cavities. These first appear in the inner nuclear layer (INL) by P12. The histology of the XLRS rat model consistently shows apparently normal retinal development through age P10, as judged by normal inner retinal cellular lamination by light microscopy. But at P12 age, many small intra-retinal cysts first appear in the middle of the INL adjacent to the soma of Muller glial cells (MGCs). These schisis spaces then enlarge rapidly, and by P15, they occupy the entire space of the normal INL.

We are interested in what contributes to the appearance and progression of these cysts, and we have noted that by the P10–P12 age, rod photoreceptors begin to elaborate inner and outer segments and exhibit light response activity. This age also coincides with the first eye opening at P12–P15 when the retina responds to environmental light. We had found that retinoschisin protein plays some role in photoreceptor processes even during development, as loss of retinoschisin in maturing mouse rod photoreceptors altered light-driven translocation of transducin but not arrestin between rod inner and outer segments [11]. Consequently, we wondered whether light-driven retinal activity could contribute to the appearance and progression of schisis cavities during retinal development in this XLRS rat model.

Exposure of rat pups to light even prior to eye opening, as early as the second postnatal week, can affect retinal function by evoking waves of spreading depression across the retina, and the segregation of retinal projections to the lateral geniculate nucleus of the brain [4,5,12]. Consequently, in conducting the study, we took care to have total light exclusion with XLRS rat pups even prior to conception by housing XLRS breeding pairs in continuous total 24 h darkness. This was performed in a specialized, separate vivarium facility on the campus of the University of California, Davis. Mothers and newborn pups were then kept in total darkness, and pups were weaned, segregated, and maintained in total darkness through age P31. This article is a revised and expanded version of preliminary data presented as a poster at ARVO 2025 [13].

MGCs’ involvement in XLRS pathology has been suggested previously [10,14,15], yet the precise mechanisms remain unresolved. We hypothesized that the dysregulation of water and ion channel homeostasis contributes to the early formation of schisis cavities during retinal development. Therefore, we investigated whether MGC activation and soma swelling are involved in schisis formation at postnatal day 12 (P12).

## 2. Materials and Methods

### 2.1. Animals

The research was carried out in compliance with ARVO’s animal use guidelines for Ophthalmic and Vision Research. The animal study protocol was approved by the Institutional Animal Care and Use Committee (IACUC) of the University of California at Davis (protocol code #23299; date of original approval was 24 March 2021 and renewal 24 March 2023). Long Evans Rs1KO and wild-type (WT) rats (Charles River Laboratories, San Francisco, CA, USA) were used to establish a breeding colony. The *Rs1*-exon1-knockout (Rs1KO) was generated by our lab in Long Evans rats using CRISPR/Cas9 by deleting Exon 1, with assistance from Horizon Discovery (Saint Louis, MO, USA) [10]. The breeding colony is maintained at UC Davis Teaching and Research Animal Care Services (TRACS) animal husbandry facility. Using our protocol, genotyping was performed using rat toe clippings at Transnetyx, Inc. (Cordova, TN, USA) for Rs1 status. For dark rearing (D/D), mating pairs were continuously raised in total darkness, with no exposure to light, in a specialized vivarium at UC Davis. In dark-rearing rooms, rats were housed in a controlled environment for temperature and humidity. To guarantee complete darkness, the animal room had a prep room where personnel would enter, turn off the lights, and then open the animal room door in complete darkness. Lab personnel checked the temperature and humidity daily, using red lights for maintenance activities.

### 2.2. Ocular Tissue Collection and Processing

Rats were sacrificed by asphyxiation with carbon dioxide. Eyes were oriented, enucleated, and fixed with 97% methanol/3% glacial acetic acid for up to 5 days before embedding in paraffin [16]. Sagittal sections 5 µm thick were cut through the eye and stained with hematoxylin and eosin (H&E) (Modified Harris Hematoxylin, 72711, Richard-Allan Scientific LLC, Kalamazoo, MI, USA; Eosin Y disodium salt, E4382-25G, Sigma-Aldrich, Inc., St. Louis, MO, USA). Retinal images were collected using a Nikon Eclipse e800 microscope with a DS-Ri1 digital camera (Nikon, Tokyo, Japan). Dark-reared animals were sacrificed in total darkness, while retina slices were prepared under regular lighting conditions. Retinas from standard cyclic light/dark rearing (L/D) and fully dark-reared, dark/dark (D/D) postnatal (P) day 12 (P12), P15, and P30 Rs1KO and WT littermate rats were collected and examined via histology and immunohistochemistry following previously published procedures [10].

### 2.3. Histology and Immunohistochemistry

Tissue sections for morphology examination were deparaffinized in xylene and rehydrated, followed by hematoxylin and eosin (H&E) staining. Paraffin sections for immunohistochemistry were rehydrated before blocking and then washed with phosphate buffer 0.1% Tween 20 (1x PBST) and preincubated with serum (5% normal goat serum, or 10% normal donkey serum, and 0.1% Tween 20 in 1x PBS) at RT for 2 h. Primary antibodies were added at the appropriate dilution using a blocking buffer containing 1x PBS and incubated overnight (Table 1).

Tissue sections were washed with 1x PBST 3 times, 15 min each, followed by secondary antibody labeling. The fluorescent secondary antibodies (Alexa Fluor 488 goat anti-mouse, Alexa Fluor 568 goat anti-rabbit, Alexa Fluor 488 goat anti-chicken, Alexa Fluor 647 goat anti-mouse, and Alexa Fluor 647 goat anti-guinea pig; Invitrogen, Carlsbad, CA, USA) were added to retinal sections at a 1:1000 dilution in PBST and incubated for 1.5 h (Table 2).

Tissue sections were washed in 1x PBST 3 times for 15 min each, and then cover-slipped with DAPI Fluoromount-G^®^ (Cat#: 0100-20, SouthernBiotech, Birmingham, AL, USA) mounting media and imaged on the Olympus Fluoview FV3000 Confocal Laser Scanning Microscope (Olympus, Tokyo, Japan). Preliminary studies investigated the expression of Vimentin (Vim), ionized calcium-binding adaptor molecule 1 (Iba1), and glial fibrillary acidic protein (GFAP) to establish optimal methods for ocular tissue collection, sectioning, and processing before the final studies. At the final fluorescent labeling stage, 36 slides (three biologic replicas for each genotype, WT, and Rs1KO, at three ages, P12, P15, and P30, and repeated set for both dark and light rearing) were deparaffinized together, washed, and incubated overnight with antibody aliquots from the same master mix.

### 2.4. Measurement of Cavity Size

H&E-stained sections were imaged on the microscope and analyzed in ImageJ version 1.54h, https://imagej.net/ij/ (accessed on 16 February 2024). The cavity regions were isolated and selected using the “Polygon Selection Tool,” and all other regions were removed. A threshold was then applied to isolate the cavities. Cavity size was measured using the “Analyze Particles” function in ImageJ, with the “outline” option enabled to confirm that the desired cavities were accurately selected. 

### 2.5. Statistical Analysis of Cavity Size

Data were analyzed using Prism 8 (GraphPad Software, La Jolla, CA, USA) and presented as mean ± SEM. Group comparisons were made with an unpaired *t*-test with Welch’s correction. A *p*-value of less than 0.05 was considered significant.

### 2.6. Study Design

A total of 12 breeding animals (6 males and 6 females) were used, consisting of 3 breeder pairs per rearing condition: light/dark (L/D) and dark/dark (D/D). Each pair comprised a heterozygous female (X^Rs1KO^/X^WT^) and a wild-type male (X^WT^/Y). From these breeding pairs, hemizygous male littermates (X^Rs1KO^/Y and X^WT^/Y) were collected and sacrificed at three developmental time points: P12, P15, and P30. At each time point, n = 3 animals (6 eyes) per genotype per group were analyzed, yielding 36 animals and 72 eyes. To minimize litter-specific and environmental confounders, three independent breeding pairs were established for each rearing condition (L/D and D/D), and litters were selected from across breeders to ensure distributional balance across groups. This pilot study was designed to test a binomial hypothesis for the presence or absence of cavity formation under D/D-rearing conditions. Assuming the null hypothesis (H_0_) corresponds to a 0% occurrence rate, the minimum sample size required to detect at least one “Yes” outcome (cavity formation) with 95% power and α = 0.05 depends on the true rate under the alternative hypothesis (H_1_): for a 10% true rate, 29 animals; for a 50% true rate, 5 animals; and for a 100% true rate, 1 animal. In our study, cavity formation was observed in 100% of animals under D/D rearing, for which the minimum required sample size is 1. Accordingly, our final experimental design included 36 animals (72 eyes), exceeding the minimum requirement and providing robust support for our binary outcome hypothesis. Sample size calculations were performed using the Binomial Reliability Demonstration Test calculator “https://reliabilityanalyticstoolkit.appspot.com/sample_size (accessed on 20 May 2025)”.

## 3. Results

### 3.1. Tiny Schisis Cavities Begin to Form in the INL of Dark-Reared Animals by P12

**Figure 1:**
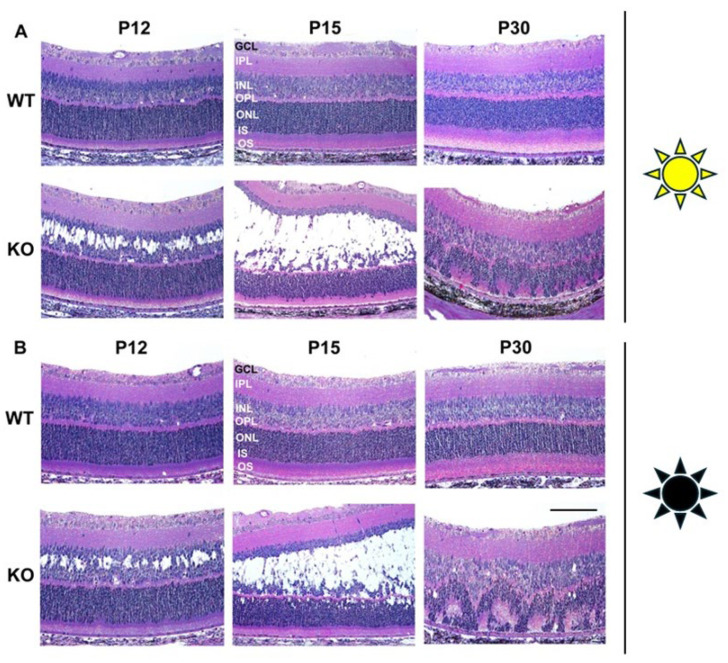
Retinal morphological alterations of Rs1KO rat model at P12, P15, and P30. (**A**) Histology of WT and Rs1KO (KO) retinas in normal lighting conditions (L/D). At the earliest age examined (P12), small cavities appeared in the inner nuclear layer (INL) of the KO. The cavities continued to increase rapidly with age. Cells from the outer nuclear layer (ONL) were found to be displaced into the inner segment (IS)/outer segment (OS) by P15. At P30, the cavities disappeared, and a few ONL cells were still present with the outer plexiform layer (OPL) showing signs of disturbance. (**B**) Histology of WT and KO retinas of dark-reared animals (D/D). Similar morphological alterations were observed in the dark-reared animals at the examined time points in KO. Ganglion cells (GCL); inner plexiform layers (IPL); inner nuclear layer (INL); outer plexiform layers (OPL); outer nuclear layers (ONL); inner segments/outer segments (IS/OS); retinal pigment epithelium (RPE) (scale bar: 50 μm).

The retinal morphology of light/dark (L/D)- and dark/dark (D/D)-reared animals was analyzed with H&E staining at P12, P15, and P30 of Rs1KO and WT (Figure 1). By P12, the Rs1KO rat model exhibited small, discernible intra-retinal schisis cavities within the central region of the INL. These cavities enlarged rapidly over the next three days, and by P15, they significantly altered the structural integrity of the INL [10].

### 3.2. Dark Rearing Does Not Reduce Cavity Formation Versus Standard Cyclic Lighting

**Figure 2:**
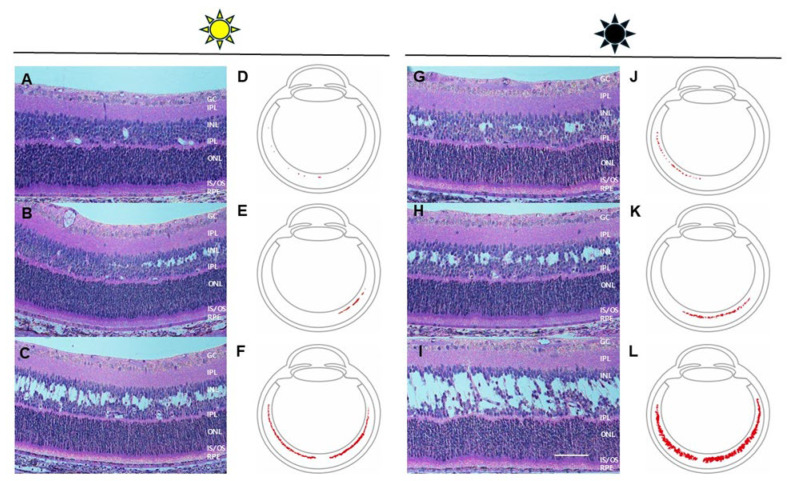
(**A**–**C**) Representative H&E-stained sections of Rs1KO, P12 retinas (20×) under light rearing (L/D), and (**G**–**I**) representative H&E-stained sections (20×) under dark rearing (D/D), showing an overall increase in cavity size in the D/D compared to the L/D. (**D**–**F**) Overall cavity distribution is depicted using ImageJ analysis for L/D rearing and (**J**–**L**) for D/D rearing, indicating somewhat more extensive cavities in D/D than in L/D rearing (scale bar: 50 μm). Ganglion cells (GC); inner plexiform layers (IPL); inner nuclear layer (INL); outer plexiform layers (OPL); outer nuclear layers (ONL); inner segments/outer segments (IS/OS); retinal pigment epithelium (RPE).

Cavities were present at P12 for all Rs1KO rats, independent of whether reared in darkness (D/D) or in light (L/D) (Figure 2, Table 3). Dark rearing did not delay cavity appearance, and, contrary to our expectations, schisis cavities were not smaller in dark-reared XLRS rats; in fact, they appeared larger. We then confirmed that the machinery for light activation was present in all animals at P12 by looking at the histological presence of rod outer segments (ROSs) and the presence of rhodopsin (see text below).

### 3.3. Immune Cells (Iba and GFAP) Distribution and Activation in Dark vs. Light Rearing

**Figure 3:**
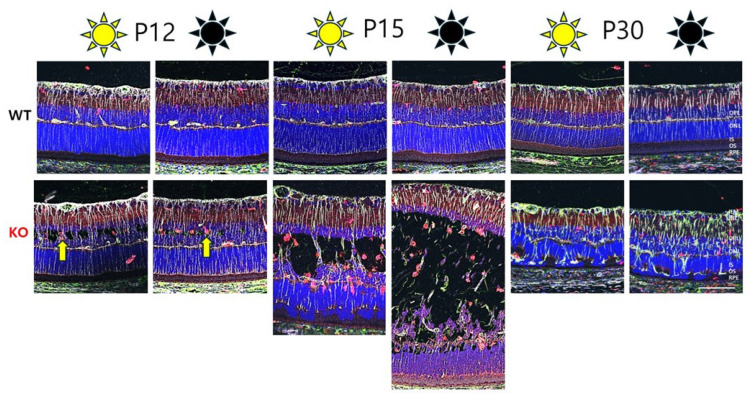
Comparative analysis of retinal morphology of Rs1KO and WT rats reared under dark (D/D) vs. light (L/D) conditions. Retinal morphology is similar in the retinal samples between light- and dark-reared animals at P12, P15, and P30: Vimentin (silver); Iba1 (red); GFAP (green); DAPI (blue); Yellow arrow points at Iba1^+^ microglia. Ganglion cells (GC); inner plexiform layers (IPL); inner nuclear layer (INL); outer plexiform layers (OPL); outer nuclear layers (ONL); inner segments/outer segments (IS/OS); retinal pigment epithelium (RPE), (scale bar: 100 μm).

**Figure 4:**
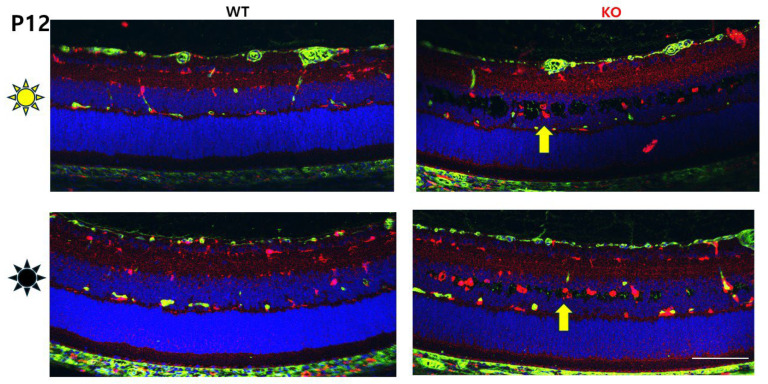
Glial and macrophage activity at P12 in Rs1KO in dark vs. light rearing. At P12, Iba1^+^ microglia (yellow arrow) increased in RS1KO retinas in both dark- and light-reared retinas. Iba1 (red); DAPI (blue) (scale bar: 100 μm).

We also investigated whether the immunologic status was comparable for D/D- and L/D-reared rats. A panel of retinal markers was used to examine whether differential immune cell activation might contribute to cavities in D/D-reared animals, using Vimentin (Vim) labeling as a marker of MGC fibers, and Iba1 and GFAP as markers of cellular activation. Iba1 (ionized calcium-binding adaptor molecule 1), also known as allograft inflammatory factor 1 (AIF1), is a well-established marker for the activation of microglia and macrophages [17]. Glial fibrillary acidic protein (GFAP) is an intermediate filament (IF) III protein found in astrocytes and MGCs as an indicator of tissue stress, and it has been associated with retinal degeneration [18]. Figure 3 shows representative images of P12, P15, and P30 for WT and Rs1KO animals, as well as for both L/D- and D/D-reared animals. By P12, when schisis appeared, Iba1^+^ cells had accumulated in regions of schisis. Iba1^+^ cells were more abundant in Rs1KO animals than WT animals and appeared slightly more prevalent in D/D- than L/D-reared Rs1KO animals (Figure 3 and Figure 4). At P30, Iba1^+^ cells were more prevalent in the retina of D/D-reared Rs1KO animals than L/D-reared (Figure 3, P30 panels).

GFAP expression at P12 was only seen at the level of ganglion cells and intermingled astrocytes. However, by P15, Müller cell fibers were also positive for GFAP, as indicated by the overlapping expression of Vimentin (a marker for MGC fibers) and GFAP. By P30, GFAP expression in MGC fibers was much stronger in Rs1KO than in WT, irrespective of D/D or L/D rearing.

### 3.4. Photoreceptor Rhodopsin Expression at P12

**Figure 5:**
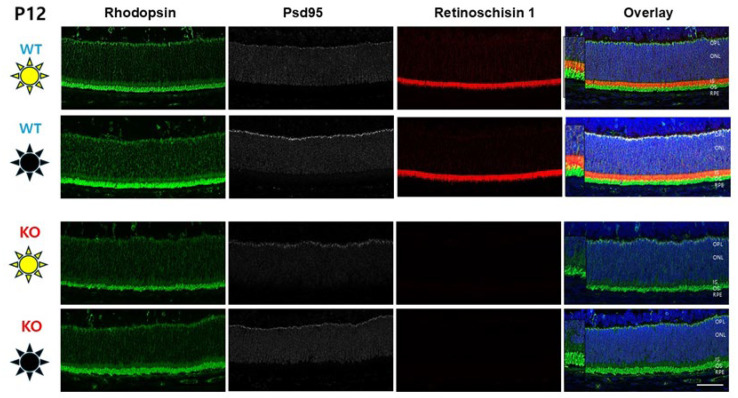
Photoreceptor inner- and outer-segment length in dark vs. light rearing. Photoreceptor structure at P12 was verified in D/D- and L/D-reared animals. Lack of retinoschisin (Rs1—red) protein does not affect rod structure, as imaged by rhodopsin (Rho—green) in rod outer segments (ROSs) and PSD95 (silver) at the synapse. The ROSs were slightly longer (insets) in dark-reared rats for both WT and Rs1KO animals (scale bar: 50 μm).

This study is preconditioned on the suitable development of photoreceptors by P12 in both D/D- and L/D-reared mice. Photoreceptor response to light requires rhodopsin expression in the outer segments, and this begins by about P5. We examined the presence of rhodopsin in the D/D- and L/D-reared P12 animals, using rhodopsin and retinoschisin fluorescent antibody labeling. Rod outer segments (ROSs) and inner segments (RISs) appeared equally developed in Rs1KO and WT animals in L/D- and D/D-reared conditions (Figure 5) (noting that Rs1KO animals do not express Rs1). Consistent with the previous report [19], by Rho labeling, the ROSs for both WT and Rs1KO appeared slightly longer at P12 for D/D-reared animals. There was no apparent change in the RISs.

## 4. Discussion

### 4.1. Eye Opening and Light Exposure

Eye opening and light exposure are important for retinal development and differentiation, and this event coincides with numerous physiological responses, including synapse and neuroglial maturation [20,21,22]. Light exposure alters the metabolic state of photoreceptors and downstream neurons [23,24], thereby increasing the demand for ionic regulation and energy metabolism. A key player in this process is Na^+^/K^+^-ATPase, which maintains ion gradients essential for retinal function [25]. Retinoschisin (Rs1), a protein deficient in the XLRS mouse model, is known to bind the β2 subunit of Na^+^/K^+^-ATPase, stabilizing this complex and supporting structural integrity and proper synaptic signaling. In the absence of Rs1, as in Rs1KO mice, this interaction is disrupted, potentially compromising the ATPase’s ability to maintain ion homeostasis during heightened activity associated with light exposure and post-eye opening maturation [25,26,27]. In the XLRS mouse model, retinoschisin deficiency induces persistent aberrant waves of activity, affecting neuroglial signaling in the inner retina at a young developmental age [28]. The waves were associated with glutamatergic neurosignaling in the proximal retina and bursts of activity in MGCs. This abnormal neuronal transmission appeared around the time of eye opening and cystic schisis formation, suggesting that while light and neuronal activity escalate ionic demands, the underlying vulnerability might be from disrupted RS1–Na^+^/K^+^-ATPase signaling.

As early retinal structural schisis cavity formation in XLRS animals occurs at P12–P15, overlapping with eye opening [29], this generated our question of whether eye opening and light exposure were involved in cavity formation. If the light was a trigger for the development of cavities, one would expect that cavity formation for Rs1KO pups raised in darkness would lag behind or even be nonexistent compared to same-age rat pups raised under normal cyclic lighting conditions. However, that was not the case. Rather, retinal cavities in the D/D- and L/D-reared conditions were the same at P12. Indeed, in this small sample of animals, if anything, the cavities of D/D-reared Rs1KO animals were possibly bigger compared to L/D-reared controls.

Light is known to affect cavity size: XLRS mice reared in low vivarium lighting (20 lux) had fewer and smaller cavities and better lamellar organization of the inner retina at 4 months old compared to others reared in moderate light (300 lux) [1]. However, this effect was not noted for younger age cohorts: XLRS mice at 1 month age (i.e., which is closer to the rat developmental P12 stage of the present study) had similar retinal schisis cavity extent between the D/D and L/D groups [1], indicating a different effect of light on developmental activity in the present study than the mature retina of 4-month-old XLRS mice.

Light exposure and dark adaptation influence retinal morphology and function in Rs1KO mice. It was reported at the ARVO 2025 meeting that larger cysts are detected by OCT in the morning after whole-night dark adaptation, while smaller cysts were found in the evening after entire-day light exposure [30]. This phenomenon may be related to higher activity of fluid removal mechanisms during light exposure, thus shrinking the cavities. The retina expels fluid primarily through active transport across the retinal pigment epithelium (RPE) and passive hydrostatic and oncotic forces. Active transport is crucial for maintaining retinal function, while passive forces assist when the RPE barrier is compromised [31].

### 4.2. Diurnal Variation in XLRS Cavities

Recent clinical studies have demonstrated that schisis cavities in X-linked juvenile retinoschisis (XLRS) exhibit significant diurnal variation. Specifically, patients with XLRS show increased central foveal thickness in the morning, which gradually decreases throughout the day, with the most pronounced changes occurring between 9 a.m. and 1 p.m. [1,2]. This pattern suggests that the size of schisis cavities is not static but fluctuates over the course of the day.

According to our results, light does not appear to be the determining factor for the opening of the schisis. This result implies that the timing of the first retinal schisis cavity formation is developmentally programmed. It appears to be a developmental step in which the presence of retinoschisin is essential for the stability and structural preservation of the retina. As retinoschisin is not present in Rs1KO animals, the retinal tissue splits. 

### 4.3. Microglial Activation in XLRS Retina

Microglia are resident immune cells in the retina that play a role in maintaining retinal homeostasis. These cells become activated in pathological conditions, adopting pro- or anti-inflammatory phenotypes. Microglia activation in XLRS retinas is associated with the release of pro-inflammatory cytokines and chemokines, which can further exacerbate retinal damage. The increased number of Iba1^+^ cells alongside retinal cavities in XLRS suggests a robust microglial response to retinal damage even as early as P12 (Figure 3 and Figure 4).

Studies in animal models of XLRS have shown that microglial activation occurs early in the disease process. In the Rs1^−/Y^ rat model, microglial activation is evident by P7, preceding significant photoreceptor degeneration [10]. The early activation of Iba1 indicates pathological processes even before overt schisis cavities begin to form at P12.

The increased number of Iba1+ cells in the developing XLRS retina highlights the potential of targeting microglial activation and inflammation as a therapeutic strategy. Gene augmentation therapy reduced microglial activation and inflammation in XLRS [32]. Rs1 expression in the Rs1^−/Y^ rat model, achieved by applying AAV8-Rs1 at P5-6, rescued the inner nuclear layer (INL) and outer plexiform layer (OPL) cavity formation and attenuated microglial activation, suggesting that restoring homeostasis by restoring Rs1 function served to reduce inflammation [10]. Understanding the molecular mechanisms underlying these processes will facilitate the development of effective therapies.

## 5. Conclusions

- Retinoschisis cavities formed at the same time in dark- and light-reared XLRS rats, indicating that the timing and extent of schisis formation are not driven by the metabolic activity of light exposure but apparently by developmental events.
- In our small sample, cavity size tended to be larger under dark-rearing conditions than under cyclic light/dark rearing, possibly due to reduced RPE fluid pumping activity that is normally stimulated by light.
- Dark-reared Rs1KO and WT rats have longer photoreceptor OSs (previously reported by Matt LaVail [19]) as RPE phagocytosis is decreased in the dark in adult rodents, allowing for ROS elongation in the absence of shedding of the distal tip.
- These results highlight the complexity of XLRS pathology, but we found no evidence that light-driven metabolic activity accounted for schisis cavity formation.

## Figures and Tables

**Table 1:** The primary antibodies (Abs) and dilution used in fluorescent immunohistology.

*Primary Antibody*	*Species*	*Vendor*	*Cat#*	*Dilution*
*Vimentin (Vim)*	Chicken	Custom Ab, Fitzgerald Lab	n/a	1:1000
*Iba1*	Rabbit	Wako Chemicals, Richmond, VA, USA	019-19741	1:500
*GFAP*	Mouse	Cell Signaling Technology, Inc. Danvers, MA	36705	1:500
*Rhodopsin (Rho)*	Mouse	Santa Cruz Biotech	sc-57432	1:500
*Psd-95*	Rabbit	Cell Signaling Technology, Inc. Danvers, MA	34505	1:500
*Retinoschisin 1 (RS-1)*	Guinea Pig	Custom Ab, Sieving Lab	n/a	1:1000

**Table 2:** Fluorescent secondary Abs and dilutions used in fluorescent immunohistology.

*Secondary Antibody*	*Vendor*	*Cat#*	*Dilution*
*Goat anti-chicken, AF488-conjugated*	Invitrogen, Carlsbad, CA, USA	A-32931	1:1000
*Goat anti-mouse, AF488-conjugated*	Invitrogen, Carlsbad, CA, USA	A-10667	1:1000
*Goat anti-rabbit, AF568-conjugated*	Invitrogen, Carlsbad, CA, USA	A-11011	1:1000
*Goat anti-mouse, AF647-conjugated*	Invitrogen, Carlsbad, CA, USA	A-21235	1:1000
*Goat anti-guinea pig, AF647-conjugated*	Invitrogen, Carlsbad, CA, USA	A-21450	1:1000

**Table 3:** Comparison of cavity size in Rs1KO rats at P12 under light (L/D) and dark (D/D) rearing. Statistical analysis and a graphic view of the data are presented below.

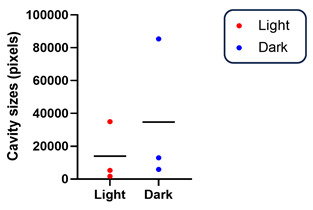			
**Light-Rearing (L/D)**		**Dark-Rearing (D/D)**	
	**Cavity Size (Pixels)**		**Cavity Size (Pixels)**
A	1741	**G**	5860
B	5263	**H**	12,979
C	34,970	**I**	85,401
**Mean Values of Cavity Size in Rs1KO Rats Under L/D and D/D**			
	**Light-Rearing (L/D)**	**Dark-Rearing (D/D)**	***p*-Value**
Mean ± SEM	13,991 ± 10,538	34,747 ± 25,410	0.511

## Data Availability

The original contributions presented in this study are included in the article. Further inquiries can be directed to the corresponding author.
